# A mixed-methods study describing behavioral factors that influenced general practitioners’ experiences using triage during the COVID-19 pandemic

**DOI:** 10.1186/s12875-021-01469-x

**Published:** 2021-07-03

**Authors:** Shaun Lackey, Kelly Ann Schmidtke, Ivo Vlaev

**Affiliations:** 1 Freelance GP and Clinical Director at NHS North Tyneside Clinical Commissioning Group, North Shields, England; 2 University of Warwick, Medical School Building, Coventry, CV4 7HL England; 3 University of Warwick, Business School, Coventry, England

**Keywords:** Digital triage, General practice, Telephone triage, Theoretical domains framework, Triage

## Abstract

**Background:**

Early in the COVID-19 pandemic, general practices were asked to expand triage and to reduce unnecessary face-to-face contact by prioritizing other consultation modes, e.g., online messaging, video, or telephone. The current study explores the potential barriers and facilitators general practitioners experienced to expanding triage systems and their attitudes towards triage during the COVID-19 pandemic.

**Method:**

A mixed-method study design was used in which a quantitative online survey was conducted along with qualitative interviews to gain a more nuanced appreciation for practitioners’ experiences in the United Kingdom. The survey items were informed by the Theoretical Domains Framework so they would capture 14 behavioral factors that may influence whether practitioners use triage systems. Items were responded to using seven-point Likert scales. A median score was calculated for each item. The responses of participants identifying as part-owners and non-owners (i.e., “partner” vs. “non-partner” practitioners) were compared. The semi-structured interviews were conducted remotely and examined using Braun and Clark’s thematic analysis.

**Results:**

The survey was completed by 204 participants (66% Female). Most participants (83%) reported triaging patients. The items with the highest median scores captured the ‘Knowledge,’ ‘Skills,’ ‘Social/Professional role and identity,’ and ‘Beliefs about capabilities’ domains. The items with the lowest median scores captured the ‘Beliefs about consequences,’ ‘Goals,’ and ‘Emotions’ domains. For 14 of the 17 items, partner scores were higher than non-partner scores. All the qualitative interview participants relied on a phone triage system. Six broad themes were discovered: patient accessibility, confusions around what triage is, uncertainty and risk, relationships between service providers, job satisfaction, and the potential for total digital triage. Suggestions arose to optimize triage, such as ensuring there is sufficient time to conduct triage accurately and providing practical training to use triage efficiently.

**Conclusions:**

Many general practitioners are engaging with expanded triage systems, though more support is needed to achieve total triage across practices. Non-partner practitioners likely require more support to use the triage systems that practices take up. Additionally, practical support should be made available to help all practitioners manage the new risks and uncertainties they are likely to experience during non-face-to-face consultations.

**Supplementary Information:**

The online version contains supplementary material available at 10.1186/s12875-021-01469-x.

## Background

Before the COVID-19 pandemic, the default mode for general practice consultations in the United Kingdom was largely face-to-face, with practices triaging to other modes in particular cases or when demand overwhelmed capacity. Early in the COVID-19 pandemic, the need to socially distance increased, and practices were asked to adapt to “total triage” wherein all patients’ needs would be assessed before their consultation and non-face-to-face consultation modes would be prioritized, e.g., telephone and video/online [1]. Things changed rapidly. From January 2020 to April 2020, the percentage of consultations conducted face to face dropped from 80 to 47%, and the percentage conducted via telephone or video/online rose from 14 to 49%. These changes remained relatively stable through August 2020, when the current study was conducted [2]. The current study examines the behavioral factors that influence the use of triage systems during the COVID-19 pandemic.

While total triage can be accomplished in many ways, the National Health Service (NHS) England published a four-stage total triage model aligning with its ambitious Long-Term Plan [1, 3]. In stage 1, patients type their information into a structured online form or call the practice to tell a receptionist what to enter into that form. In stage 2, staff filter requests and send queries to appropriate practitioners. In stage 3, practitioners review each patient’s file to select the most appropriate consultation mode: online messaging, video, telephone, or face-to-face. In stage four, the consultation is conducted, during which an additional consultation may be recommended via a new mode. While stage 3 is most clearly the point at which the mode of the consultation is selected, strengthening all four stages is important to optimize the NHS total triage model in practice.

While the NHS total triage model is relatively straightforward conceptually, its real-world application is not. For example, some clinical commissioning groups have made a digital triage software available to their practices. That software is mainly offered based on budgetary and administrative factors rather than on practice preferences or usability factors. What triage system practices adopt is largely decided by the general practitioners who are partners (practice owners), and they may not consider non-partner views (salaried staff). For more information about this distinction please see Oxtoby (2019) [4]. Several approved digital software systems are available, e.g. AskmyGP and eConsult, but funding may not be sufficient for practices to select software outside that made available by their clinical commissioning group [5–7]. At the time of the current study, AskMyGp was used by over 200 general practices [8]. Research conducted in 2017 found that, while some practices and patients benefited from adopting AskMyGp, there was not sufficient evidence to recommend its scale and spread [9].

Rather than adopting a digital triage system, practices may simply expand existing systems that promote social distancing, such as the telephone first approach. The telephone first approach requires that all patients consult with a general practitioner on the phone first and only come to the practice if their concerns cannot be resolved by phone. This flips the triage model, such that triage occurs after an initial telephone consultation. In a review of 150 practices adopting telephone first, face-to-face consultations decreased about 38% [10]. However, achieving this decrease requires greater management of staff time and patient expectations than practitioners expect [11]. The current study does not aim to compare telephone first and digital triage systems. Both systems can be used to increase social distancing during pandemics. Both systems have been described here to help the reader appreciate two widely used options general practices may adopt, and the confusions that may arise, as they expand their use of triage and reduce unnecessary face-to-face contact.

To understand the behavioral factors that influence whether partner and non-partner practitioners use triage, the present study uses the Theoretical Domains Framework: the leading framework for identifying key barriers and facilitators of behavior change [12]. The validated version of the Theoretical Domains Framework condenses 112 unique theoretical constructs described by 33 theories of change into 14 domains, including: ‘Knowledge,’ ‘Skills,’ ‘Social/Professional role and identity,’ ‘Beliefs about capabilities,’ ‘Optimism,’ ‘Beliefs about consequences,’ ‘Reinforcement,’ ‘Intentions,’ ‘Goals,’ ‘Memory attention and decision processes,’ ‘Environmental context and resources,’ ‘Social influences,’ ‘Emotions,’ and ‘Behavioral regulation.’ [13] In addition to being linked to theory, each domain is linked to empirically supported techniques best suited to leverage its underlying concepts, which are described by the evolving Theories and Techniques Tool [14, 15].

The current study aims to understand practitioners’ experiences using triage during the COVID-19 pandemic in a way that can promote further use of triage. The first objective is to quantitatively describe behavioral factors that influence practitioners’ triage use. We compare the experiences of partner and non-partner practitioners to explore their unique support needs. The second objective is to capture practitioners’ more nuanced experiences with and attitudes toward triage using qualitative interviews.

## Methods

### Design

A mixed-method research design was used, as recommended by the Medical Research Council [16]. The quantitative and qualitative research components were conducted concurrently (in August and September 2020) to offer a more comprehensive understanding than either method alone would offer [17]. The quantitative survey was the dominant method and supported by the qualitative method. The quantitative survey allows a quick assessment of a fixed set of empirically and theoretically informed factors for many participants. The qualitative interviews allow for a more nuanced exploration of non-fixed factors for a small number of participants. The results for each component were reviewed by two authors, SL and KAS. The results of each method were independently analyzed and are presented independently to appreciate their unique contributions. SL and KAS discussed the integration of themes across methods and agreed to present the integrated findings in the discussion, where potential improvement strategies are explored.

### Quantitative methods

#### Survey recruitment

The research team aimed to recruit at least 200 general practitioners from the United Kingdom. As this was an exploratory study, this sample size was informed by feasibility factors and rules of thumb rather than formal calculations [18]. The survey was initially emailed to general practitioners working in the North East region of England, starting with key contacts located in each of the following clinical commissioning groups: North Tyneside, Northumberland, and Newcastle and Gateshead. An invitation was also posted on WhatsApp Messenger and Facebook. Participants were encouraged to forward the survey to other practitioners, and the resultant sample is a sample of convenience.

#### Survey content

The anonymous online survey was designed in Typeform®. An initial draft of the survey was reviewed by one general practitioner external to the research team to ensure it was easy to understand and could be completed in less than 10 min. As this is a new survey designed for an exploratory study, the survey has not been validated, and validating the survey was not one of the study’s objectives. Before advancing to the survey items, participants viewed an information sheet explaining the purpose and scope of the study and indicated their informed consent.

Participants responded to three groups of items, see Supplemental Materials 1. The first group of items asked about participants’ background characteristics, including gender, age, job status (partner or non-partner), and clinical commissioning group. The second group of items asked whether they had experience using a telephone triage system or a digital triage system. If they had experience using a system, they then indicated on a 7-point Likert scale, in which 1 indicated lower levels, how using that system influenced their stress levels, time management, and job satisfaction compared to using no triage system.

The third group of items asked about the behavioral factors that may influence practitioners’ triage use according to the Theoretical Domains Framework, see Table 1. These items were informed by Huijg et al.’s (2014) validated template survey to capture all 14 behavioral factors, called “domains” [19], as has been done in previous studies aiming to understand the barriers and facilitators to smoking cessation and general practitioners’ recommendations of self-directed exercise [20, 21]. Most domains were captured with a single item. For most items, participants expressed agreement with a statement using a 7-point Likert scale, in which 1 indicated strong disagreement, 4 indicated a neutral response, and 7 indicated strong agreement. For the items about ‘Intentions’, ‘Goals, and ‘Emotions’ participants indicated how strong their intentions were, how often they felt distracted, and how nervous they felt, respectively, using similar 7-point scales. Lastly, participants were asked to provide their email address if they were willing to take part in a follow-up interview.

**Table 1:** Theoretical domains captured by survey items

Theoretical Domain	Item
Knowledge(1)	I am aware of the objectives of patient triage.
Knowledge(2)	I am familiar with how to triage patients in general practice.
Skills	I have the skills required to triage patients in general practice.
Social/Professional role and Identity	I feel it is my responsibility as a general practitioner to triage patients effectively.
Beliefs about capabilities(1)	I am confident that, if I wanted, I could triage patients effectively within general practice.
Beliefs about capabilities(2)	For me, triaging patients effectively in general practice is very easy.
Optimism	I feel that patient triage is the best way to match the patient’s problem with the right person, for the right amount of time.
Beliefs about consequences(1)	For me, triaging patients effectively in general practice is very useful.
Beliefs about consequences(2)	If I triage my patients in general practice, it will have disadvantages for my relationship with my patients.*
Reinforcement	If I triage my patients in general practice, I feel I am making a difference.
Intentions	How strong is your intention to triage your patients in primary care over the next year?
Goals	Generally, how often do you feel something else takes priority over setting up patient triage systems?*
Memory, attention, and decision processes	When I need to concentrate on triaging my patients in general practice, I have no trouble focusing my attention.
Environmental context and resources	Patient triage systems provide the possibility to adapt services to the patient’s needs.
Social influences	I can rely on my colleagues when things get tough in regard to patient triage in general practice.
Emotion	Thinking about yourself and how you normally feel as a professional that delivers patient care, to what extent do you feel nervous with regard to patient triage within general practice?*
Behavioral regulation	I have a clear plan under what circumstances I will triage my patients in general practice.

* reverse-scored item

#### Analyses

Analyses were conducted in SPSS (v.26). Negatively worded items were reverse scored. Then, the median responses (and interquartile ranges) for each item were calculated. The median scores are interpreted relatively, such that higher scores indicate domains that are stronger facilitating factors, and lower scores indicate domains with greater room for improvement. Next, for each of the Theoretical Domains Framework items, the same calculations were performed for partner and non-partner practitioners separately. Their responses were compared using Mann-Whitney U tests, rather than independent samples T-tests because the data were non-parametric. The alpha value was set at 0.05 to assess significant differences. Due to the exploratory nature of the study, no corrections were made for multiple comparisons.

### Qualitative methods

#### Interview recruitment

The research team aimed to interview at least 12 participants, as data saturation for broad themes is likely to be reached by this point [22]. To recruit participants, an email was sent to survey participants who indicated their willingness to take part, i.e., convenience sampling. The email included additional information participants should consider, e.g., that their interview would take approximately 15-min and be audio recorded. A time was then scheduled with participants who continued to express interest. At the beginning of the interview, participants affirmed their consent to participate.

#### Interview materials

The interviews followed a semi-structured format with principle and probe questions, see Table 2. The topic guide was designed to encourage 15-min conversations. Participants were encouraged to talk freely about each question.

**Table 2:** Interview Topic Guide

Type	Question
Principle	Tell me how you use triage in your current day-to-day work and any past experience you may have had?
	What are your thoughts on patient triage? Specifically, what are the advantages or disadvantages?
	How do patients feel about triage, i.e. “patient satisfaction”?
	How do you feel triage impacts on your stress, workload, time management or job satisfaction?
	Do you have any thoughts or opinions regarding triage or digital triage that we haven’t covered and you would like me to capture today?
Probe	What is your experience of referring on to other [non-face-to-face] services within triage?
	Do any patient groups have particular difficulties with triage models?
	What do you think could make triage better?
	Do you have any specific thoughts on total digital triage?
	Do you think that your practice will carry on using total triage after COVID-19?

#### Interview analyses

The interviews were transcribed and initially analyzed by a single researcher (SL), using Braun and Clarke’s inductive thematic approach [23]. SL is a general practitioner who was also experiencing a transition to total triage at the time of this study. A strength of SL’s position is that it allowed a more intuitive understanding of the words participants used to describe their experiences. A limitation is that such intuitions may be colored by personal experiences. After reading each interview, any concept coded multiple times was identified as a potential theme. After reading all transcripts, the transcripts were re-examined to be certain no data were missed. The themes were then further refined with a co-author (KAS), who is an academic behavioral scientist with no experience working in general practice and could increase the accessibility of themes for readers who are not practitioners. This refinement process helped to abstract the minimum number of broad themes that described the concepts discussed.

## Results

### Quantitative findings

#### Participants

The survey was completed by 204 eligible participants (66% female), most of whom were between the ages of 40 and 49 (43%). As it is unknown how many practitioners were invited to take up the survey, a participation rate could not be calculated. Most participants worked for clinical commissioning groups located in the North East region of England (*N* = 123), but the other 8 English regions were represented (*N*s = 4–18), as were Scotland (*N* = 4), Wales (*N* = 4), and Northern Ireland (*N* = 1); 18 participants did not provide their clinical commissioning group. More participants identified as partner (*N* = 134) than non-partner (*N* = 68) practitioners; 1 participant did not disclose and is not included in the statistics comparing partner to non-partner practitioners. Thirty-four participants said that they had not experienced using either triage system, 9 used only a digital system, 119 used only a telephone triage system, and 42 used both.

#### Triage systems

The participants who used the telephone triage system believed that it had little influence on their stress levels (Mdn = 4, IQR = 2) but had a slightly negative effect on their job satisfaction (Mdn = 3, IQR = 2) and had a slightly positive effect on their time management (Mdn = 5, IQR = 3). The participants who used the digital triage system believed that it had little effect on their stress levels (Mdn = 4, IQR = 3) and job satisfaction (Mdn = 4, IQR = 3) but had a slightly positive effect on their time management (Mdn = 5, IQR = 3).

#### Behavioral factors

Table 3 shows each domain item surveyed alongside the number of participants responding. The median responses (and interquartile range) are displayed for participants’ overall and then for partner and non-partner practitioners separately. The median scores were high (six out of seven) for the domain items about ‘Knowledge,’ ‘Skills,’ ‘Social/Professional role and identity,’ and ‘Beliefs about capabilities’. There was more room for improvement in other domains, with median scores being four, a neutral response, for the domain items about ‘Beliefs about consequences,’ ‘Goals,’ and ‘Emotions.’ Descriptively, partner practitioners’ responses were higher than non-partners for all items, with significant differences occurring for 12 of the 17 items.

**Table 3:** Surveyed domains alongside the number of participants, and the median responses overall and for partner and non-partner practitioners separately

Theoretical Domain	Number	Median (Interquartile Range)			Mann-Whitney Test			
		Overall	Partner	Non-partner	Partner mean rank	Non-partner mean rank	Z-value	*p*-value
Knowledge(1)	204	6 (1.75)	6 (1)	6 (2)	106.5	93.25	1.62	0.11
Knowledge(2)	203	6 (1)	6 (1)	6 (2)	105.53	93.55	1.47	0.14
Skills	204	6 (1.75)	6 (1)	6 (2)	110.7	85.11	3.12	0.00*
Social/Professional role and Identity	204	6 (2)	6 (2)	6 (2)	103.82	98.46	0.65	0.52
Beliefs about capabilities(1)	204	6 (1)	6 (1)	6 (2)	110.39	85.71	3.04	0.00*
Intentions	204	6 (2)	6 (2)	5 (2)	113.66	79.36	4.06	0.00*
Beliefs about capabilities(2)	204	5 (2)	5 (2)	4 (2)	110.48	85.54	2.92	0.00*
Optimism	204	5 (2)	6 (1)	5 (2)	112.44	81.73	3.61	0.00*
Beliefs about consequences(1)	203	5 (1)	6 (2)	5 (1)	108.8	87.43	2.54	0.01*
Reinforcement	204	5 (2)	5 (2)	4 (1)	109.03	88.34	2.44	0.01*
Memory, attention, and decision processes	203	5 (3)	5 (2)	4 (2)	108.72	87.59	2.48	0.01*
Environmental context and resources	204	5 (1.75)	5 (1)	5 (2)	107.44	91.43	−1.9	0.06
Social influences	204	5 (2)	5 (2)	5 (3)	109.6	87.24	−2.62	0.01*
Behavioral regulation	203	5 (2)	6 (3)	4 (3)	111.73	81.78	−3.52	0.00*
Beliefs about consequences(2)	204	4 (3)	4 (3)	4 (1)	103.22	99.64	0.42	0.68
Goals	204	4 (1)	4 (2)	3 (1)	107.85	90.63	2.03	0.04*
Emotion	204	4 (2)	4 (3)	4 (1.5)	93.15	119.19	−3.05	0.00*

* *p*-value < 0.05

### Qualitative findings

#### Participants

At the end of their survey, 48 participants expressed willingness to be interviewed, and 15 responded to the invitation. Interview times were scheduled with 14 participants. However, as only 1 of these participants solely used a digital triage system, it was determined that a more reliable analysis could be accomplished by focusing on the remaining 13 participants who relied on a telephone triage system. Six participants identified as male and seven as female. All identified as partner practitioners, whom our survey findings suggest may require less support to use the triage systems their practice adopts than non-partner practitioners. Most interview participants worked in the North East region (*N* = 10): The remaining three participants worked in London, Yorkshire, and Scotland. This may limit the generalizability of our findings across practitioner roles and locations. The following six broad themes were discovered.

#### Theme 1: patient accessibility

Participants believed that the number of patients accessing practices had increased. Such beliefs were likely, in part, due to the initial decrease at the beginning of the first lockdown, followed by an increase once new ways of accessing practices were put in place. Some participants noted that triaging empowered their practice: “*to see all the patients trying to seek help together and prioritize them outside of the queue of presentation.”* However, if demand kept increasing, many were concerned their practice may not cope well. One participant reflected that the number of patients accessing their practice was growing. This participant commented that:

“*There are no barriers at all, and we are a free service that is highly valued. In the end, we end up rationing by our waiting times and queuing … and I’m just worried about where this is going.*”

New ways of accessing the practice also created new challenges for vulnerable patients who did not have supportive carers, e.g., patients with difficulties hearing. Participants raised concerns about a lack of dedicated methods to reach some vulnerable populations, e.g., people who are homeless. One participant noted that:

*“I think it [our triage] excludes most vulnerable people that need the access— if you’re not online or haven’t got a phone, you’ve got a hearing problem, or you’ve got dementia, mental health issues, or learning disabilities, or you’re socially awkward. I just think it’s putting lots of barriers in those patients’ way, and some of them are actually really needy, and nobody seems to be interested in that. Nobody is talking about that … some of the most needy people they can’t deal with the triage process, so they get lost in the system.”*

#### Theme 2: confusions around what triage is

While triage systems have the potential to meet patient needs faster, that did not seem to happen. Many participants reflected that telephone triage felt too much like telephone consultation. One participant commented that their triage practice was: “*not really triage—it’s just an emergency consultation*.” Another reflected that: “*I thought that total telephone triage would save a lot of time, but it surprises me actually how time-consuming some of the conversations can be*.” If the practice has adopted the telephone first approach, addressing patient concerns first and then triaging to another appointment mode for unmet needs makes sense. However, practitioners still must decide when to transition from offering a supportive consultation service to offering a supportive triage service. Alternatively, if the practice has not adopted the telephone first approach, then practitioners must decide when to transition from a triage service to a consulting service. Either way, this transition may prove difficult for many. One participant felt that it was:

*“quite hard to manage the expectations of the patient and say, ‘ok that’s your problem then we’re going to book you into an appointment in a week to discuss this properly.’ It is quite hard because the patients’ expectation of what the triage contact is about maybe is not the same as what triage really is [about].”*

If a practice is not clear about what triage model they adopt, then different practitioners may operate under different mental models, potentially increasing confusion and decreasing efficiency. One participant commented that change was particularly difficult during the COVID-19 pandemic because they were unable to have a group meeting. This participant said that:

*“I guess change is the hardest thing to do in GP [general practice], for us all, and we’re not all at the same page at the same time in terms of making a change to the system, and its quite hard. It’s an effort to bring everyone to that point. Even harder when we can’t all sit in the same meeting room as a mass group to talk about things.”*

#### Theme 3: risk and uncertainty

Participants believed that time must be given to triage in order to reduce risks associated with triaging patients to an inappropriate consultation mode. For instance, if a patient contacts the practice with concerns about something that requires a blood test, triaging them to attend a telephone consultation would be inappropriate. There was a sense that inappropriately triaging to a face-to-face mode was more common than inappropriately triaging to a non-face-to-face mode because the face-to-face mode felt less risky. Overall, participants were concerned that they might miss something important if they did not see a patient in person. As part of a strategy to minimize risk, participants agreed that it is: “*better to have a senior person doing the initial triage [who is] able to make a decision and take responsibility.*” This senior person could model best practices for other practitioners.

Another risk discussed involved practitioners not being able to access familiar “*subtle body language or non-verbal*” signals to explore potential problems. One participant commented that the video appointment mode did not help them to access these visual signals, saying that:

“*Video doesn’t work near us. The internet is not reliable on either our side or their side, which pixelates and freezes all the time and that is worse than having a phone call.*”

Participants did not mention alternative non-visual signals they might use during non-face-to-face consultations to explore potential problems, e.g., changes in vocal tones or unexpected background noise. Participants were also concerned about the brief duration of calls. They believed that a longer appointment was often needed to build sufficient trust with patients, as this trust enables patients to express deeper problems. For example, one participant recollected that:

*“Patients present with minor ailments because they really have something else going on. We see it all the time, don’t we? They come and they sit there. They test you with something really boring and you think, ‘Why did they come with a cough?’, … and then they tell you, ‘Actually, doctor, I just want to let you know, that my husband’s been beating me up,’ or ‘Actually, doctor, I just want to let you know, I’m really low and I’ve had these horrible suicidal thoughts,’ and it [these sorts of comments] always comes at the end. Or, you know, a male patient often is quite embarrassed about a male [problem], you know, impotency for example. They always tell you at the end, don’t they, of a long consultation? They have to gain your trust.”*

#### Theme 4: relationships between service providers

Participants recognized the benefits of having cooperative relationships between service providers. One participant noted that: “*lots of things come to doctors that might not need to, but it requires good accessibility from other services.*” Another mentioned that:

“*Mental health services are down. We don’t have access to all the investigations that we need [e.g., from hospital services], and we can’t examine patients. So, I think the stress is building up from the fact that we can’t actually do what we’d like to do for our patients, and we can’t signpost them appropriately—there are no other services.*”

As the pandemic lifts and other services resume, these difficulties may naturally resolve where good relationships previously existed, but there was a sense that problems due to poor relationships pre-date the pandemic. Participants highlighted a longstanding issue with the general practice being used as a backstop expected to pick up the tab when other services ran out of capacity or were unresponsive to patient needs. One participant reflected that their practice was trying to overcome this problem by bringing in a partner physiotherapist. However, when that physiotherapist was unavailable, patients still expected the practice to offer treatment.

#### Theme 5: job satisfaction

Triage had varied effects on practitioners’ job satisfaction. Some expressed positive views, e.g.: *“I would never want to go back to what I had before, where you don’t know what is coming through the door.”* Another reflected that: *“I enjoy that kind of work because actually, it is quite varied … I quite like the challenge of what goes where … I quite like the decision-making.”* Some negative views were also captured, but most negative views blurred the lines between what is triaging and what is consulting. For example, one participant stated that:

“*I know that a lot of job satisfaction comes from good interactions with patients. So, if our aim is always not to see people, that might reduce [job satisfaction]. I certainly don’t want to feel like I’m working in a call center. That’s what I’d be worried about*.”

Thus, the negative views discovered in this theme are strongly connected to the confusions discussed in Theme 2.

#### Theme 6: digital triage potential

The potential effectiveness of digital triage partially depended on patients’ ability to provide succinct and uncomplicated information on the digital triage interface. Therefore, realizing the potential benefits of digital triage will require creating more user-friendly interfaces. For example, one participant commented that digital triage is: “*quite useful for people asking very specific things—people asking about medication or wanting the extension of a sick note or something like that*.” For patients with less straightforward problems the digital triage process was often a barrier to accessing more traditional and conversational appointment modes. These conversations are often needed to figure out what the problem is, and to empower patients to use more specific terms the next time they interact with the practice. The digital triage system could be modified to push patients with less straightforward issues, or language difficulties, to a more conversational appointment mode faster.

Generally, participants felt that this technology was here to stay, and they wanted a more streamlined approach to use it efficiently. One participant reflected that digital triage has “*been around since the early noughties,”* but that they did not *“think anyone has found a good sustainable way of doing it.*” Another commented that “*We wouldn’t go back to the old way of working now,”* but later continued that if this move was to be successful *“then maybe some practical training would be needed.”*

## Discussion

To the authors’ knowledge, this is the first paper describing the behavioral factors that influenced general practitioners’ experiences with and attitudes towards triage during COVID-19. Most of our practitioners (83%) had experience using a triage system, and the use of a telephone triage system was more common than a digital triage system. The quantitative findings suggest that there is room for improvement across three domains: ‘Beliefs about consequences,’ ‘Goals,’ and ‘Emotions.’ The quantitative findings also suggest that non-partner practitioners may require more support than partner practitioners in order to embrace the triage model their practice adopts. The qualitative findings revealed a more nuanced perspective regarding partner practitioners’ concerns, what worked well, and what needed to be addressed to optimize triage systems.

Described now are some behavior change techniques that the Theories and Techniques Tool recommends for optimizing triage. For instance, the survey picked out ‘Beliefs about consequences,’ and the interviews suggested that participants wanted to see examples of best practice. If a repository of examples is created, the Theories and Techniques Tool also recommends providing information about the practical and emotional consequences experienced by those implementing successful triage systems (techniques 5.1, 5.3, and 5.6) [15]. Practitioner “Goals” could be supported by asking them to set a goal around triage and to review their progress towards it (techniques 1.1, 1.3, 1.5, 1.6, and 1.7) [15]. The interview findings suggest that setting time-sensitive goals may be particularly important to help practitioners prioritize the time they think is needed to conduct triage well.

Lastly, regarding ‘Emotions,’ the interviews indicated that participants felt concerned about additional risk and uncertainties arising in non-face-to-face consultations, in part because they cannot see their patients. To reduce negative emotions, practitioners could be provided with advice (technique 11.2) [15] For example, during telephone consultations some concerns could be addressed if patients using smartphones could send photographs of their ailments. With proper informed consent patients can send photographs, but many practitioners likely require additional training before they feel comfortable requesting, receiving, and storing photographs properly [24]. Additional training may also be required that helps doctors use non-visual strategies to recognize and explore instances of potential abuse [25]. The interview participants did not seem to be aware of non-visual cues they could use.

One of the difficulties with implementing triage seems to be understanding what triage is and how it is distinct from a consultation. In a narrow sense, the word ‘triage’ has been used to describe a process for determining the order with which patients are treated, based on condition severity or likelihood of recovery [26]. The concept of ‘total triage’ is broader, such that triage entails determining not only what patients to see first but also the mode through which their scheduled consultation will take place. An underlying assumption of the NHS total triage model is that identifying the best mode will help practitioners keep up with increasing patient demand. In theory, this is possible but realizing that possibility becomes more difficult as the number of potential modes increases and the opportunity for identifying the wrong mode increases.

Our interview participants expressed some confusion regarding when the triage process ends and when the consultation process begins. To optimize triage partner practitioners must be transparent about the model they adopt. Any confusions they experience are likely experienced to a greater extent by non-partner practitioners. In many models, triage comes before the consultation. For example, in the NHS total triage model, a video consultation itself is not triage, rather a patient may be triaged to attend a video consultation. In contrast, in the telephone first approach, a telephone consultation comes first, and then patients may be triaged to attend a future consultation via a new mode to resolve unmet concerns. Compared to the NHS total triage model, the telephone first approach may offer a simpler mental model to latch onto, because the initial consultation mode chosen is the same every time. However, while the telephone first approach can serve many patients’ needs, some patients experience obvious problems, e.g., people with conditions that require examinations or people who do not have telephone access during their work shifts [27, 28]. In these circumstances, a digital triage system may be able to spot patients who would be better served via a different consultation mode without prior direct clinical interaction. If done well, digital triage has the potential to increase patient wellbeing and practitioner job satisfaction.

Several limitations of our study are now noted. Regarding the study’s external validity, the participants involved in the survey and interviews were largely recruited from clinical commissioning groups in the North East region of England, and therefore the results may not generalize to other regions [29]. Regarding internal validity, although the current study was informed by a validated template survey, the survey itself was not validated and was only piloted on a single practitioner external to this research team. Also, a larger percentage of survey participants, 43%, were between the ages 40 and 49 than would be anticipated based on NHS Digital’s census, in which 29% of general practitioners are between 40 and 49 [30]. Practitioners 50 years and greater (30% of the workforce based on the census) and those 39 or less (40%) may have different experiences.

Another limitation is that, while the interviews certainly clarify some survey results, the connections between the methods are not always clear. Future studies using mixed-methods could make these links clearer, e.g. by analyzing interviews using a deductive coding method to draw out themes according to the same framework used in the survey [31] or by using a joint-display pillar integration technique to synthesize finding across components with equal dominance [32]. While multiple researchers refined the themes, a single researcher coded the transcripts. Future studies may use multiple coders for the transcripts to increase the reliability of resultant codes and themes. The COREQ checklist may serve as a helpful checklist to guide the conduct of future multi-method studies [33].

## Conclusions

The use of triage rapidly expanded in general practices following the COVID-19 pandemic. While positives of the triage system were noted, many practitioners expressed frustration regarding precisely how triage should be implemented. For total triage to be successful, partner and non-partner practitioners need to understand how the process can work best to deliver wins for both patients and providers. Further research is required to guide the development of successful triage tools and training systems. The current study identified several behavioral factors, including ‘Beliefs about consequences,’ ‘Goals,’ and ‘Emotions,’ that future quality improvement interventions should address. What techniques are selected and how they are implemented will need to be tailored to meet each practice’s needs.

## Supplementary Information

**Additional file 1:.** Supplemental Materials 1. Survey Items

## Data Availability

The datasets used and/or analyzed during the current study are available from the corresponding author on reasonable request.

## Abbreviations

NHS: National Health Service
