# Supplementary material for: A mixed-methods study describing behavioral factors that influenced general practitioners’ experiences using triage during the COVID-19 pandemic
Source: BMC Fam Pract. 2021 Jul 3;22:146. doi: 10.1186/s12875-021-01469-x (PMC8254621; doi:10.1186/s12875-021-01469-x)
Supplement: Supplementary file 1 — Additional file 1:. Supplemental Materials 1. Survey Items [file 12875_2021_1469_MOESM1_ESM.docx]

A mixed-methods study describing behavioral factors that influenced general practitioners’ experiences using triage during the COVID-19 pandemic

Shaun Lackey [shaunlackey@nhs.net](mailto:shaunlackey@nhs.net)^1^

*Kelly Ann Schmidtke [Kelly.A.Schmidtke@warwick.ac.uk](mailto:Kelly.A.Schmidtke@warwick.ac.uk)^2^

Ivo Vlaev [Ivo.Vlaev@wbs.ac.uk](mailto:Ivo.Vlaev@wbs.ac.uk)^3^

^1^ Freelance GP and Clinical Director at NHS North Tyneside Clinical Commissioning Group

^2^ Warwick Medical School, Medical School Building, Coventry CV4 7HL

^3^ Warwick Business School, University Of Warwick, Scarman Rd, Coventry CV4 7AL

* Corresponding Author: Kelly Ann Schmidtke. [Kelly.A.Schmidtke@warwick.ac.uk](mailto:Kelly.A.Schmidtke@warwick.ac.uk) 07758 933026. Warwick Medical School, Medical School Building, Coventry CV4 7HL

Supplemental Materials 1. Survey Items

**[BACKGROUND]**

1. Are you Male or Female please? (Male, Female, Prefer not to say, Other),

2. May I ask how old are you? (less than 30, 30-39, 40-49, 50-59, 60+),

3. What is your current Job role? (GP partner, salaried GP, Locum GP, GP trainee, F2 trainee),

4. What CCG do you work in please? (North Tyneside, Newcastle and Gateshead, Northumberland, Other (: free-text space))

5. Are you currently working in a GP practice which uses a *telephone triage* model? (Yes, No)

**[STRESS, TIME MANAGEMENT, AND JOB SATISFACTION]**

*If YES, then continue to items 6-8. If NO, then skip to item 9.*

6. Compared to non-triage, how does telephone triage affect your *stress levels*? (1 = less stress to 7 = more stress),

7. Compared to non-triage, how does telephone triage affect your *time management*? (1 = worse to 7 = better),

8. Compared to non-triage, how does telephone triage affect your *job satisfaction*? (1 = less satisfied to 7 = more satisfied)

9. Are you currently working in a GP practice which uses a *total digital triage* model?

*If YES, then continue to items 10-12. If NO, then skip to item 13.*

10. Compared to non-triage, how does total digital triage affect your *stress levels*? (1 = less stress to 7 = more stress),

11. Compared to non-triage, how does total digital triage affect your *time management*? (1 = worse to 7 = better),

12. Compared to non-triage, how does total digital triage affect your *job satisfaction*? (1 = less satisfied to 7 = more satisfied)

**[THEORETICAL DOMAINS FRAMEWORK]**

13. I am aware of the objectives of patient triage. (1 = strongly disagree to 7 = strongly agree)

14. I am familiar with how to triage patients in general practice. (1 = strongly disagree to 7 = strongly agree)

15. I have the skills required to triage patients in general practice. (1 = strongly disagree to 7 = strongly agree)

16. I feel it is my responsibility as a general practitioner to triage patients effectively. (1 = strongly disagree to 7 = strongly agree)

17. I am confident that, if I wanted, I could triage patients effectively within general practice. (1 = strongly disagree to 7 = strongly agree)

18. For me, triaging patients effectively in general practice is very easy. (1 = strongly disagree to 7 = strongly agree)

19. I feel that patient triage is the best way to match the patient's problem with the right person, for the right amount of time. (1 = strongly disagree to 7 = strongly agree)

20. For me, triaging patients effectively in general practice is very useful. (1 = strongly disagree to 7 = strongly agree)

21. If I triage my patients in general practice, it will have disadvantages for my relationship with my patients. (1 = strongly disagree to 7 = strongly agree)

22. If I triage my patients in general practice, I feel I am making a difference. (1 = strongly disagree to 7 = strongly agree)

23. How strong is your intention to triage your patients in primary care over the next year? (1 = not at all strong to 7 = very strong)

24. Generally, how often do you feel something else takes priority over setting up patient triage systems? (1 = not very often to 7 = very often)

25. When I need to concentrate on triaging my patients in general practice, I have no trouble focusing my attention. (1 = not at all strong to 7 = very strong)

26. Patient triage systems provide the possibility to adapt services to the patient's needs. (1 = not at all strong to 7 = very strong)

27. I can rely on my colleagues when things get tough in regard to patient triage in general practice. (1 = not at all strong to 7 = very strong)

28. Thinking about yourself and how you normally feel as a professional that delivers patient care, to what extent do you feel nervous with regard to patient triage within general practice? (1 = not at all nervous to 7 = very nervous)

29. I have a clear plan under what circumstances I will triage my patients in general practice. (1 = not at all strong to 7 = very strong)
